# Beyond the Randomized Controlled Trial: A Review of Alternatives in mHealth Clinical Trial Methods

**DOI:** 10.2196/mhealth.5720

**Published:** 2016-09-09

**Authors:** Quynh Pham, David Wiljer, Joseph A Cafazzo

**Affiliations:** ^1^ Institute of Health Policy, Management and Evaluation Dalla Lana School of Public Health University of Toronto Toronto, ON Canada; ^2^ Centre for Global eHealth Innovation Techna Institute University Health Network Toronto, ON Canada; ^3^ Centre for Addictions and Mental Health (CAMH) CAMH Education Toronto, ON Canada; ^4^ Faculty of Medicine Department of Psychiatry University of Toronto Toronto, ON Canada; ^5^ Institute of Biomaterials and Biomedical Engineering Faculty of Medicine University of Toronto Toronto, ON Canada

**Keywords:** mobile health, mobile applications, smartphones, medical informatics, research design, clinical trials

## Abstract

**Background:**

Randomized controlled trials (RCTs) have long been considered the primary research study design capable of eliciting causal relationships between health interventions and consequent outcomes. However, with a prolonged duration from recruitment to publication, high-cost trial implementation, and a rigid trial protocol, RCTs are perceived as an impractical evaluation methodology for most mHealth apps.

**Objective:**

Given the recent development of alternative evaluation methodologies and tools to automate mHealth research, we sought to determine the breadth of these methods and the extent that they were being used in clinical trials.

**Methods:**

We conducted a review of the ClinicalTrials.gov registry to identify and examine current clinical trials involving mHealth apps and retrieved relevant trials registered between November 2014 and November 2015.

**Results:**

Of the 137 trials identified, 71 were found to meet inclusion criteria. The majority used a randomized controlled trial design (80%, 57/71). Study designs included 36 two-group pretest-posttest control group comparisons (51%, 36/71), 16 posttest-only control group comparisons (23%, 16/71), 7 one-group pretest-posttest designs (10%, 7/71), 2 one-shot case study designs (3%, 2/71), and 2 static-group comparisons (3%, 2/71). A total of 17 trials included a qualitative component to their methodology (24%, 17/71). Complete trial data collection required 20 months on average to complete (mean 21, SD 12). For trials with a total duration of 2 years or more (31%, 22/71), the average time from recruitment to complete data collection (mean 35 months, SD 10) was 2 years longer than the average time required to collect primary data (mean 11, SD 8). Trials had a moderate sample size of 112 participants. Two trials were conducted online (3%, 2/71) and 7 trials collected data continuously (10%, 7/68). Onsite study implementation was heavily favored (97%, 69/71). Trials with four data collection points had a longer study duration than trials with two data collection points: *F*_4,56_=3.2, *P*=.021, η^2^=0.18. Single-blinded trials had a longer data collection period compared to open trials: *F*_2,58_=3.8, *P*=.028, η^2^=0.12. Academic sponsorship was the most common form of trial funding (73%, 52/71). Trials with academic sponsorship had a longer study duration compared to industry sponsorship: *F*_2,61_=3.7, *P*=.030, η^2^=0.11. Combined, data collection frequency, study masking, sample size, and study sponsorship accounted for 32.6% of the variance in study duration: *F*_4,55_=6.6, *P*<.01, adjusted *r*^2^=.33. Only 7 trials had been completed at the time this retrospective review was conducted (10%, 7/71).

**Conclusions:**

mHealth evaluation methodology has not deviated from common methods, despite the need for more relevant and timely evaluations. There is a need for clinical evaluation to keep pace with the level of innovation of mHealth if it is to have meaningful impact in informing payers, providers, policy makers, and patients.

## Introduction

With over 165,000 mobile health (mHealth) apps on the Apple App Store and Google Play Store catalogues and 3 billion downloads in 2015 alone [1], mHealth apps represent a mature, robust marketplace for a new generation of patients who seek patient-empowered care and mHealth publishers who aim to facilitate this practice. mHealth apps are currently being developed for many different clinical conditions including diabetes [2], heart failure [3], and cancer [4], and have the potential to disrupt existing health care delivery pathways.

In recent years, numerous calls have been made to address the challenges inherent in mHealth app evaluation [5-7]. Key barriers were identified by researchers at the National Institutes of Health mHealth Evidence Workshop, notably the difficulty of matching the rapid pace of mHealth innovation with existing research designs [8]. Explicit attention was drawn to the randomized controlled trial (RCT), which has long been considered the primary research study design capable of eliciting causal relationships between health interventions and consequent outcomes [9]. However, RCTs are notoriously long—the average duration of 5.5 years from enrollment to publication clearly risks app obsolescence occurring before study completion [10]. With high-cost trial implementation and a rigid protocol that precludes mid-trial changes to the intervention in order to maintain internal validity, RCTs are perceived as an incompatible, impractical evaluation methodology for most mHealth apps [11-15]. There is also an inherent quality of software that does not lend itself to the rigidity of the RCT—software is meant to change, evolve, progress, and learn over time, all at a rapid pace. Rigid trial protocols undermine this principle attribute, since controlled trials were designed for interventions that take years, even decades to develop, that is, medical devices and drugs. In concluding the mHealth Evidence Workshop, researchers identified the need to develop novel research designs that can keep up with the lean, iterative, and rapid-paced mHealth apps they seek to evaluate.

The Chicago-based Center for Behavioral Intervention Technologies has endeavored to design methodological frameworks that can appropriately support mHealth evaluation. Mohr and colleagues proposed the Continuous Evaluation of Evolving Behavioral Intervention Technologies (CEEBIT) framework as an alternative to the gold-standard RCT [16]. The CEEBIT methodology is statistically powered to continuously evaluate app efficacy throughout trial duration and accounts for changing app versions through a sophisticated elimination process. The CEEBIT also thoughtfully addresses many other RCT-specific considerations, from randomization to inclusion/exclusion criteria to statistical analysis.

Additional alternatives to the RCT have also been presented, including interrupted time-series, stepped-wedge, regression discontinuity, and N-of-1 trial designs that may limit interval validity but are more responsive and relevant for evaluating mHealth interventions [8]. Novel factorial trial designs have been proposed for mHealth research and are increasingly being used to test multiple app features and determine the optimal combinations and adaptations to build an effective app. These include the multiphase optimization strategy (MOST) [17], the sequential multiple assignment randomized trial (SMART) [18], and the microrandomized trial [19]. Suggestions have also been made on how to increase the efficiency of traditional RCTs themselves, including using within-group designs, fully automating study enrollment, random assignment, intervention delivery and outcomes assessment, and shortening follow-up through modeling long-term outcomes [13]. Further, best practice evaluation methods in the field of human-computer interaction, notably usability testing and heuristic evaluation, have been widely adopted in mHealth research and are well suited to assess the efficacy of user-driven, digitally operationalized behavioral mechanisms required to elicit stable changes in health outcomes [20-22]. These alternatives allow us to reconsider the RCT for a more flexible and iterative evaluation approach that will mimic the attributes of software-based behavioral interventions and their agile app development process, where it is acceptable and preferable to learn from a poor trial outcome sooner in order to redesign the intervention more quickly and subsequently show success sooner.

In parallel to the development of novel research designs like the CEEBIT, new industry initiatives have also introduced novel platforms to deploy mHealth evaluations. In 2015, Apple announced the release of ResearchKit, a software framework designed for health research to allow iPhone users to participate in research studies more easily [23]. ResearchKit allows for the digital collection of informed consent, a process that has historically hindered the accrual of patients into trials and the scalability of clinical research. It also enables access to real-time data collected from the iPhone’s accelerometer, gyroscope, microphone, and global positioning system (GPS), along with health data from external wearables (eg, FitBit, Apple Watch) to gain real-time insight into a participant’s health behaviors [24]. Evidence of ResearchKit’s impact can already be seen in several Apple-promoted research trials deployed for a range of conditions [25-27]. It is not difficult to imagine ResearchKit being adapted for use as a tool to evaluate mHealth app efficacy—an app claiming to help patients self-manage their diabetes could be launched using the ResearchKit framework and evidenced for efficacy through sensor data and in-app surveys.

Given the development of alternative evaluation methodologies and the launch of novel technologies to automate mHealth research, we sought to determine if these initiatives were being implemented in current clinical trials. Through this review, research designs and methods for current mHealth clinical trials were identified and characterized in an effort to understand the views of the field toward novel frameworks for evaluating mHealth apps.

## Methods

A review of the ClinicalTrials.gov registry was conducted in November 2015 to identify and examine current clinical trials involving mHealth apps. The following search terms were trialled in a scoping search to optimize the search strategy: mobile application, mobile heath app, mobile health application, mobile app, smartphone application, and smartphone app. A Boolean search was then conducted with all these search terms combined (“mobile application” OR “mobile heath app” OR “mobile health application” OR “mobile app” OR “smartphone application” OR “smartphone app”.) However, upon comparing the search results generated from all scoping searches, the search term “mobile application” independently yielded a higher number of results compared to the Boolean search. A precautionary decision was made to use “mobile application” as the sole search term to retrieve relevant trials registered between November 19, 2014, and November 19, 2015—a 1-year period before this review was initiated. The titles and abstracts of retrieved trials were assessed for inclusion, followed by a complete review of the entire trial registration. Following the final identification of trials to include in our review, we conducted a reverse search of each trial to determine whether it would have been found through our initial Boolean search and concluded that a small number of relevant studies would have been omitted. We therefore recommend the use of “mobile application” as the preferred comprehensive search term for those looking to duplicate our search strategy.

All trials were included if they (1) evaluated mHealth apps, (2) measured clinical outcomes, and (3) were deployed exclusively on a mobile phone as a native app and not a Web-based app. Trials were excluded if (1) they evaluated mHealth apps that solely received text messages (short message service [SMS] or multimedia messaging; this was done due to a large amount of existing trials for SMS-based interventions in the literature) or phone calls as their primary behavior change modification, (2) the mHealth app was a secondary intervention or the study mixed mobile and non-mobile interventions, (3) the mHealth app was solely an appointment reminder service, and (4) the mHealth app did not require user input through active or passive (sensor) data entry.

Following the identification of studies that met inclusion criteria, trial data were extracted from the ClinicalTrials.gov website and coded according to relevant outcome variables. All data were collected directly from the registry, where trial information was originally reported and categorized by the investigators conducting the trials. Extracted data measures included trial identification, app name, study purpose, trial sponsor, targeted condition, data collection duration, data collection points, study duration, sample size, study type, control and masking methods, random allocation, group assignment, study site, qualitative components, app availability, and study design. Table 1 lists all measures that were manually coded into categories from extracted data alongside their codes. A differentiation was made in coding “data collection duration,” defined as the amount of time allotted for primary data collection as specified in the outcome measures section of each ClinicalTrials.gov record detail, and “study duration,” defined as the amount of time between initial recruitment and complete data collection as specified by the “estimated study completion date” in the trial record detail. Studies were coded as being onsite if participants had any direct face-to-face contact with a member of the research team, and online if recruitment and follow-up data collection were done remotely—if a participant was recruited in a hospital setting but follow-up data were collected through the study app, this was coded as onsite implementation. Targeted conditions were further coded into parent condition categories for analysis. All identified app titles were also searched on public app stores (ie, Apple App Store, Google Play Store) to confirm whether they were available for public download.

**Table 1:** Manually coded study variables from extracted ClinicalTrials.gov registry data.

Variable	Coded values
Study purpose	efficacy, safety/efficacy, observational
Trial sponsor	academic, industry, collaboration
Targeted condition	mental health, cardiovascular, diabetes, cancer, asthma, obesity, other
Data collection points	1-3, 4+, continuous
Sample size	0-49, 50-99, 100-499, 500+
Study type	interventional, observational
Control	standard care, active, waitlist
Masking	open, single-blind, double-blind
Group assignment	single, parallel, three groups
Study site	onsite, online
Study design	1 group pretest-posttest, 1 group posttest, 1-3 group posttest control, 2-3 group pretest-posttest control, 2-3 group posttest non-randomized control, observational

### Data Analysis

Descriptive statistics were first conducted on all variables to identify methodological data trends and parameters. In reference to Campbell and Stanley’s experimental and quasi-experimental designs for research [28], measures of whether trials collected pretest or baseline data, and also the number of data collection points throughout the trial, were recorded. This was done to identify specific study designs and assess the range of study designs deemed suitable for mHealth app evaluation.

While the focus of this review was to provide an overview of the study designs and methodologies currently being employed for mHealth research, we were also interested in exploring the relationships between methodological variables, specifically identifying potential predictor variables for study duration. We first conducted independent *t* tests and one-way independent analyses of variance (ANOVA) to determine whether there were differences in study duration for the following categorical methodological variables: study sponsorship, clinical condition, pretest data collection, data collection frequency, presence of a control group, study purpose, presence of randomization, study group assignment, qualitative data collection, and app availability. We then performed a Pearson correlation analysis to test for a correlational relationship between sample size and study duration. These preliminary analyses were conducted to determine which variables were appropriate for inclusion in a multiple linear regression analysis. The assumptions of linearity, normality, independence of errors, and homoscedasticity were met, and diagnostic tests to check for outliers, homogeneity of variance, and multicollinearity were passed. The regression was then performed with study duration as the dependent variable and all significant predictor variables from our preliminary analyses as independent variables. Extreme outlier data were excised prior to analysis, leaving a dataset that included 64 trials (90%, 64/71), each with a sample size of 500 participants or less. Statistical significance was considered at *P*<.05 unless otherwise specified. All statistical analyses were conducted using SPSS Statistics version 22 (IBM Corporation).

## Results

### General Characteristics

Of the 137 trials identified, 71 were found to meet inclusion criteria. Table 2 details each included trial and outlines their general characteristics. Key highlights include the ClinicalTrials.gov study identification, app name, target condition, sample size, and study duration.

### Methodological Characteristics

The great majority of reviewed trials were classified as interventional (96%, 68/71) with only 3 of the 71 trials (4%, 3/71) classified as observational. Most trials used an RCT design (80%, 57/71). Sixty-three of the 71 trials were classifiable under the Campbell and Stanley experimental design framework (89%, 63/71). Subdesign classifications included 36 two-group pretest-posttest control group comparisons (51%, 36/71), 16 posttest only control group comparisons (23%, 16/71), 7 one-group pretest-posttest designs (10%, 7/71), 2 one-shot case study designs (3%, 2/71), and 2 static-group comparisons (3%, 2/71). The remaining 8 trials included 2 three-group pretest-posttest control group comparisons (3%, 2/71), 1 two-group posttest non-randomized control group comparison (1%, 1/71), 1 three-group posttest non-randomized control group comparison (1%, 1/71), 1 three-group posttest control group comparison (1%, 1/71), and 3 observational studies (4%, 3/71). In total, 17 trials included a qualitative component to their methodology (24%, 17/71).

Control group assignment was divided into standard care (51%, 30/59), active treatment (44%, 26/59), and waitlist (5%, 3/59). Open masking was favored (69%, 47/68) over blinded masking (31%, 21/68). Randomization of groups was common practice among reviewed trials (84%, 57/68). There was a broad distribution of clinical conditions across the 71 trials, with mental health (17%, 12/71), cardiovascular conditions (11%, 8/71), diabetes (11%, 8/71), and cancer (10%, 7/71) leading the clinical focus. The full range of clinical conditions is shown in Table 3.

**Table 2:** General characteristics of reviewed trials registered on Cinical.Trials.gov.

ClinicalTrials.gov study ID	App name	Target condition	n	Study duration^a^
NCT02531074	Swipe Out Stroke	obesity	100	29
NCT02426814	Mobile phone app, inhaler sensor	asthma	50	6
NCT02615171	RELAX app	obesity	60	12
NCT02515500	Quitbit, digital lighter	smoking	200	21
NCT02421536	Vibrent	cancer	40	21
NCT02308176	Mobile phone app	obesity	118	12
NCT02370719	BantII	type 2 diabetes	150	25
NCT02618265	Mobile phone app	stroke	400	35
NCT02432469	Mission-2	coronary artery bypass	1000	18
NCT02429024	OneTouch Reveal, blood glucose meter	type 2 diabetes	142	12
NCT02399982	Noom Monitor	bulimia	80	27
NCT02486705	PTSD Family Coach	stress, depression, anxiety	242	8
NCT02322307	HealthPROMISE	irritable bowel syndrome	300	29
NCT02346591	Jauntly	depression, stress	298	9
NCT02503098	Recovery Record	eating disorders	12000	18
NCT02417623	OBSBIT	obesity	76	24
NCT02392000	CBT-I Coach, sleep monitor	insomnia	40	6
NCT02400710	PTSD Coach	posttraumatic stress disorder	30	32
NCT02445196	PTSD Coach	posttraumatic stress disorder	120	15
NCT02421965	FOCUS	schizophrenia	174	30
NCT02375776	CORA	cancer	104	10
NCT02451631	Health-on G, physician web monitoring	type 2 diabetes	184	11
NCT02313363	Mobile phone app	type 2 diabetes	30	3
NCT02521324	RESPERATE, breathing sensor	traumatic brain injury	40	16
NCT02501642	TBI Coach	sleeplessness	486	48
NCT02457923	M-SAKHI	malnutrition	2728	38
NCT02589730	Welltang	type 1 and 2 diabetes	234	12
NCT02431546	VIDA	coronary artery disease	40	15
NCT02359981	MyBehavior	obesity	17	1
NCT02405117	LiveWell, wrist-worn device	bipolar disorder	48	27
NCT02610894	PoCAH	surgery	40	24
NCT02472561	iHealth, Withings	peripheral artery disease	45	13
NCT02439619	TechCare	psychosis	16	35
NCT02601794	Mobile phone app	breast cancer	180	7
NCT02448888	Mobile phone app	back pain	24	11
NCT02497755	Ginger.io	anxiety, depression	25	4
NCT02555553	Noom Monitor	bulimia	200	18
NCT02554578	Mobile phone app, web platform	heart transplant	158	14
NCT02418910	KIOS-Bipolar, eMoods	bipolar disorder	50	18
NCT02510924	Airtraq	nasal obstruction, arthrosis	100	12
NCT02580396	CanADVICE+	metastatic breast cancer	25	24
NCT02350257	Mobile phone app	anxiety disorders	130	33
NCT02551640	FeatForward	type 2 diabetes	300	9
NCT02333630	AsthmaCare	asthma	200	13
NCT02588729	Pregnant+	gestational diabetes	264	38
NCT02599857	CONCOR	congenital heart disease	500	24
NCT02496728	NUYou	cardiovascular disease	800	38
NCT02565225	RheumaLive	rheumatoid arthritis	60	32
NCT02484794	Recovery Record	eating disorders	40	12
NCT02494245	STARFISH	stroke	128	24
NCT02308878	Mobile phone app	substance use dependence	65	20
NCT02592291	Mobile phone app	spinal cord and brain injuries	160	59
NCT02341235	Mobile phone app	breast cancer	120	58
NCT02470143	Mobile phone app	coronary heart disease	20	11
NCT02480062	mWELLCARE	cardiovascular disease	3600	20
NCT02477137	Mobile phone app	prostate cancer	150	40
NCT02420015	Stay Quit Coach	schizophrenia	36	20
NCT02479607	Mobile phone app	breast cancer	150	24
NCT02591459	Mobile phone app	autism	10	2
NCT02499094	Mobile phone app	depression	1004	7
NCT02382458	Mobile phone app	chronic inflammation	120	25
NCT02517047	Mobile phone app, CareTRx device	asthma	26	22
NCT02521558	Mobile phone app	Alzheimer’s disease	100	11
NCT02385643	Mobile phone app, Bluetooth sensor	alcohol dependence	100	46
NCT02317614	SteadyRx	human immunodeficiency virus	50	28
NCT02556073	MyAsthma, inhaler	asthma	112	28
NCT02302040	Team Speak	asthma	50	20
NCT02492191	Recovery Assessment by Phone Points	postoperative complications	1000	14
NCT02580409	Wellpepper	mobility limitations	76	24
NCT02341950	SCI Hard	spinal cord injury	200	12
NCT02403427	VoiceDiab, insulin pump	type 1 diabetes	42	9

^a^Study duration is measured in months.

**Table 3:** Targeted clinical conditions.

Conditions		n (%)
**Mental health**		12 (16.9)
	Anxiety	2
	Bipolar disorder	2
	Depression	1
	Psychosis	1
	PTSD	2
	Schizophrenia	2
	Stress	2
**Cardiovascular**		8 (11.3)
	Cardiovascular disease	2
	Congenital heart disease	1
	Coronary artery bypass	1
	Coronary artery disease	1
	Coronary heart disease	1
	Heart transplant	1
	Peripheral artery disease	1
**Diabetes**		8 (11.3)
	Gestational diabetes	1
	Type 1 diabetes	1
	Type 2 diabetes	5
	Type 1 and 2 diabetes	1
**Cancer**		7 (9.9)
	Breast cancer	4
	Prostate cancer	1
	General	2
Asthma		5 (7.0)
Obesity		5 (7.0)
Eating disorder		4 (5.6)
Surgery		3 (4.2)
Insomnia		2 (2.8)
Spinal cord injury		2 (2.8)
Stroke		2 (2.8)
Substance abuse		2 (2.8)
**Other**		11 (15.5)
	Alzheimer’s disease	1
	Arthritis	1
	Autism	1
	Back pain	1
	Chronic inflammation	1
	Human immunodeficiency virus	1
	Inflammatory bowel disease	1
	Malnutrition	1
	Mobility	1
	Smoking	1
	Traumatic brain injury	1

By condition in order of prevalence, 9 mental health trials were RCTs (75%, 9/12), with 4 trials designed as classic two-group pretest-posttest control group comparisons (33%, 4/12). Seven of 8 cardiovascular trials were RCTs (88%, 7/8), with all 7 designed as two-group pretest-posttest control group comparisons. Seven of 8 diabetes trials were also RCTs (87.5%; 7/8), with 5 two-group pretest-posttest control group comparisons (63%, 5/8). Most of the asthma trials were RCTs (80%, 4/5), with all 4 adhering to a two-group pretest-posttest control group comparison design. Finally, all 5 obesity trials were RCTs (100%, 5/5), but none adhered to a two-group pretest-posttest control group comparison design.

Most trials did collect pretest data prior to study implementation (68%, 46/68). Trials had on average three data collection points (mean 2.7, SD 1.2) with 7 trials collecting data continuously (10%, 7/68). Table 4 summarizes the distribution of apps across methodological variables.

**Table 4:** Distribution of apps across methodological variables.

Variable		n (%)
**Study type**		
	Interventional	68 (95.8)
	Observational	3 (4.2)
**Pretest data collected**		
	Yes	46 (67.6)
	No	22 (32.4)
**Control treatment**		
	Standard care	30 (50.8)
	Active	26 (44.1)
	Waitlist	3 (5.1)
**Masking**		
	Open	47 (69.1)
	Single-blind	17 (25.0)
	Double-blind	4 (5.9)
**Randomization**		
	Yes	57 (83.8)
	No	11 (16.2)
**Qualitative component**		
	Yes	17 (23.9)
	No	54 (76.1)
**Study location**		
	Onsite	69 (97.2)
	Online	2 (2.8)
**Data collection points**		
	One	12 (17.6)
	Two	20 (29.4)
	Three	17 (25.0)
	Four or more	12 (17.6)
	Continuous	7 (10.3)

### Descriptive Characteristics

Data collection duration was relatively short on average (median 6 months, IQR 8) with the majority of trials having a data collection period of 6 months or less (72%, 51/71). However, the range of duration was broad, with the shortest data collection period lasting 10 days and the longest period lasting 4 years.

Study duration was 20 months on average (mean 21, SD 12); researchers continued to collect secondary data for nearly a year after they had completed their primary data collection (median 12, IQR 13). This discrepancy between study duration and data collection duration was more pronounced in studies with a total duration of 2 years or more (31%, 22/71) where the average time from recruitment to complete data collection (mean 35, SD 10) was 2 years longer than the average time required to collect primary data (mean 11, SD 8). Of the 71 trials, only 7 had been completed at the time this retrospective review was conducted (10%, 7/71).

Enrollment varied across trials (median 112, IQR 158): 20 trials had a sample size of 0-49 (28%, 20/71), 10 had a sample size of 50-99 (14%, 10/71), 33 had a sample size of 101-499 (47%, 33/71), and 8 had sample sizes of over 500 participants (11%, 8/71)—the largest being 12,000 participants.

Studies with at least one component of onsite implementation were heavily favored, with 69 trials (97%, 69/71) opting for onsite recruitment and implementation. It should be noted that the trial with the largest sample size (N *=* 12,000) had online study implementation.

Nearly three-quarters of the trials (72%, 51/71) had official app names, which suggested that they were positioned for commercialization or were already available on the market. However, only 17 apps (24%, 17/71) were publicly available for download as of December 2015. Academic sponsorship was the most common form of trial funding (73%, 52/71), followed by an academic-industry collaboration (18%, 13/71) and industry sponsorship (9%, 6/71).

### Methodological Analysis

Our preliminary *t* tests and ANOVAs to determine whether differences existed in study duration across methodological variables revealed three significant variables: data collection frequency, *F*_4,56_=3.2, *P*=.021, η^2^=0.18; masking, *F*_2,58_=3.8, *P*=.028, η^2^=0.12; and study sponsorship, *F*_2,61_=3.7, *P*=.030, η^2^=0.11. Follow-up Bonferroni and Fisher’s least significant difference tests were conducted to evaluate pairwise differences among study duration means. We identified a significant difference in the means between two and four or more data collection points (mean_diff_=-15, SE=5, *P=*.025), open and single-blinded masking (mean_diff_=-10, SE=4, *P=*.026), and industry and academic study sponsorship (mean_diff_=12, SE=6, *P=*.033). Descriptive statistics for studies included in this analysis are presented in Table 5.

**Table 5:** Study duration means of trials included for analysis grouped by data collection frequency, masking, and study sponsorship.

Variable		n (%)	Mean duration (months)	SD	95% CI	
					Low	High
**Data collection points**		61 (100)				
	One	12 (19.7)	25	11	18.0	32.2
	Two	18 (29.5)	16	11	10.2	21.2
	Three	13 (21.3)	18	8	12.9	22.0
	Four or more	11 (18.0)	30	17	18.5	41.9
	Continuous	7 (11.5)	20	12	8.3	30.9
**Masking**		61 (100)				
	Open	46 (75.4)	19	11	15.2	21.8
	Single-blind	13 (21.3)	29	16	19.1	38.8
	Double-blind	2 (3.3)	16	7	-47.5	79.5
**Study sponsorship**		64 (100)				
	Academic	49 (76.6)	23	13	19.0	26.7
	Industry	5 (7.8)	10	2	7.5	13.3
	Academic-industry collaboration	10 (15.6)	15	6	10.4	19.4

A correlation analysis of the relationship between sample size and study duration revealed a positive but weak correlation between both variables: *r=*.25, *P*=.044. Based on this finding, we included sample size as a predictor variable in our multiple linear regression model for predicting study duration alongside data collection frequency (two versus four or more data collection points), masking (open versus single-blinded), and study sponsorship (academic versus industry). The focus of this analysis was prediction, so we used a stepwise method of variable entry. The results of our regression analysis indicated that all four of our predictors combined accounted for 32.6% of the variance in study duration: *F*_4,55_=6.6, *P*<.01, adjusted *r*^2^=.33. Data collection frequency alone, specifically the difference between two and four or more data collection points, was able to explain 11.5% of the variance in study duration. Together with the difference between single versus open masking, these variables explained 19.7% of the variance in study duration. Sample size added 6.7% to the explanation of variance in study duration, and the difference between academic and industry sponsorship added another 6.2%. Each step in the model added significantly to its predictive capabilities. Based on this model, the prediction equation is as follows: 13.79 + 10.71*(two versus four or more data collection points) + 6.88*(single versus open masking) + 0.04*(sample size) – 12.00*(industry versus academic sponsorship). Table 6 presents the regression coefficients and standard errors for each of the four significant predictors.

**Table 6:** Multiple linear regression model of predictors for study duration.

Variable	R^2a^	B^b^	SE_B_^c^	β^d^	*P* value
Constant		13.79	2.31		<.001
Data collection frequency (2 vs 4+ data collection points)	.12	10.71	3.68	.33	.005
Masking (single vs open-blinded)	.20	6.88	3.50	.23	.055
Sample size	.26	0.04	0.01	.31	.009
Study sponsorship (academic vs industry)	.33	-12.00	5.33	-.26	.028

^a^R^2^: amount of accounted study duration variability.

^b^B: unstandardized regression coefficient.

^c^SE_B_: standard error of the coefficient.

^d^β: standardized coefficient.

## Discussion

### Principal Findings

Our review has shown that the overwhelming majority of mHealth researchers are continuing to use the RCT as the trial design of choice for evaluating mHealth apps. The consistent use of RCTs to demonstrate efficacy across disparate clinical conditions suggests that researchers view this design to be condition-agnostic and truly the gold standard for any clinical trial evaluating app efficacy. While trials of apps for managing obesity did not adhere to a two-group pretest-posttest control group comparison design as defined by the Campbell and Stanley framework, and only a third of mental health apps used this classic RCT design, the majority of trials for other prevalent conditions did favor this specific study design to evaluate health outcomes and elicit proof of app efficacy. This homogeneity of study designs within the framework suggests that researchers are not adapting designs to align with the unique qualities inherent in the mHealth apps they are evaluating.

Some unexpected findings emerged from our review, one being the near-complete lack of variation in study implementation sites—97% of trials were conducted onsite in academic centers and hospitals, with only two trials employing online recruitment and data collection. Regarding trial duration, mHealth trials had a total data collection period of 20 months on average. We were able to identify four predictor variables that accounted for 32.6% of the variance in trial duration: data collection frequency, masking, sample size, and study sponsorship.

Our analysis of the relationship between the number of data collection points in an mHealth trial and the duration of the trial revealed that trials with four or more data collection points would have a significantly longer data collection period compared to trials with two data collection points. While this finding suggests that mHealth trials might benefit from a study implementation process that includes automated data collection through the intervention app to allow for frequent data collection without prolonging study duration, our review results are inconclusive in supporting this recommendation given the lack of a clear relationship between study length and data collection frequency. In analyzing the raw review data, there is no significant difference in study duration between one, three, and four or more data collection points, and trials with one data collection point are similarly long in duration compared to trials with four or more data collection points. With this in mind, we are cautiously optimistic in our advocacy of automated study implementation, from recruitment to data collection, for all mHealth trials.

While many trials had open masking, nearly a third chose to blind their participants or outcomes assessor, and four trials even went as far as to double-blind both participant and investigator. This level of rigor was unanticipated for a field that has been criticized for a lack of evidence demonstrating efficacy and impact [29]. We were surprised to find that single-blinded trials were significantly longer in duration compared to open trials. However, given the dearth of empirical evidence to support the role of double blinding in bias reduction [30] and the inconclusive nature of our raw data, which did not show an increase in study duration between open and double-blinded trials, more data are required to investigate this relationship prior to discounting the value of masking in favor of shorter trials.

Despite the fact that the majority of reviewed trials were funded by academic research grants, industry-academic partnerships were not uncommon and suggest that industry publishers have realized the potential of engaging with academic institutions to bolster the credibility of their apps. However, these partnerships warrant particular attention given past lessons learned from duplicitous investigative behavior exhibited by industry-funded research teams [31]. Our review results revealed that industry-funded mHealth trials were significantly shorter in duration than their academic counterparts. A potential explanation for this difference in study duration is the use of study outcomes in industry trials that are more sensitive to short-term changes (eg, quality of life, frequency of desired health behaviors, engagement with mHealth app) over outcomes with a longer trajectory towards measurable change (eg, frequency of emergency department visits, quality-adjusted life years, mortality). These trials may also be bound by competitive industry-led timelines, which dictate how long an app can spend in research and development before it must be released to generate profit—a concern that is shared but not equally prioritized in academic mHealth app development. It is apparent that industry-funded mHealth trials differ from purely academic pursuits in both research objectives and anticipated outcomes, making efforts to maintain methodological rigor and increase the transparency of industry-academic collaborations a critical endeavor as these relationships grow in popularity.

It is very clear that only a fraction of publicly available apps are evaluated [32], and our identification of 71 mHealth trials initiated over a 1-year period is in stark comparison to the tens of thousands of unevaluated apps publicly deployed during the same time period. While the mHealth trials we reviewed were methodologically rigorous, it was obvious that the methods themselves have not changed: not once in the registration of any mHealth clinical trial was the CEEBIT methodology mentioned, nor alternate methodologies that have been identified as more suitable for mHealth evaluation. The mobile phone platform on which mHealth apps are hosted is not being leveraged through initiatives like ResearchKit to improve recruitment for large sample sizes or to passively collect data with built-in sensors. This is unfortunate given the opportunity to explore and build upon mobile phone capabilities for research purposes. It was also unclear how trials with data collection periods of 2 years or more would maintain the relevance of their findings.

From our preliminary results, it appears that investigators conducting mHealth evaluations are applying positivistic experimental designs to elicit causal health outcomes. This insight is a cause for concern because it neglects to consider that (1) mHealth apps are complex interventions [33] and as such, (2) mHealth apps might therefore be fundamentally incompatible for evaluations founded on purely positivistic assumptions [34].

In addressing the first point, mHealth apps may simply be software programs on a mobile phone, but they have personal and social components that prove unstable when they are forced to be defined and controlled [35]. mHealth researchers should acknowledge that app users may intend to use technology for improved health but also exhibit unpredictable behaviors of poor compliance, deviant use, and in rare cases even negligence. This will affect both internal and external validity of traditional trials looking to prove direct causation.

To illustrate our second point, various positivistic assumptions regarding mHealth apps should be considered. A positivistic researcher might state that mHealth apps affect a single reality that is knowable, probabilistic, and capable of being objectively measured. They might think it is reasonable to make generalizable statements about the relationship between the app and consequent health outcomes. They might then assume a methodological hierarchy of research designs to validate this reality, with quantitative experimental studies being seen as the most robust, for which the RCT is the gold standard. While this viewpoint is evidently endorsed by the majority of mHealth researchers whose work was identified in this review, it has not been justified in practice due to the challenge of isolating the relationship between the user and the specific mHealth app being evaluated [14]. The hallmark of the RCT is its ability to control for contextual variables in order to only measure causal impact between independent and dependent variables. However, mHealth evaluations that implement an RCT methodology are often forced to engage in trade-offs that breach RCT protocol but increase the usage and adherence rates critical to study implementation [36]. mHealth researchers have recognized a host of research implementation barriers, from the deployment environment, to app bugs and glitches, to user characteristics and eHealth literacy [37]. It is arguably easier to prevent patients from taking a drug that might interfere with their health outcomes in a pharmaceutical trial than it is to prevent patients from using an alternative diabetes management app or reading about diabetes management strategies on a website during an mHealth trial. Finally, of the trials we reviewed, the apps we evaluated were not simple and static; they were sociotechnical systems [38] that were robust in functionality and provided timely, continuous, and adaptable care personalized to the needs of their users. If we ignore these natural attributes in evaluating apps and remain wedded to traditional research designs that view these strengths as confounders, we will fail to capture the complex technological nuances and mechanisms of change facilitated by apps [39] that can impact positive health outcomes.

### Limitations

In addressing the limitations of our review, we must acknowledge the rapid pace at which mHealth trials are being registered to ClinicalTrials.gov. In the 5 months following our initial search, 31 new trials had been added to the registry that met our inclusion criteria. On initial assessment, these trials are in line with our review findings. The majority adhere to a classic two-arm RCT trial design, target a range of complex chronic conditions, and are on average 2 years in duration. We aim to update our review in 6-month intervals to capture the high volume of incoming mHealth clinical trials.

Our study duration calculation was based on the “study start date” and “study completion date” fields reported by researchers on ClinicalTrials.gov. We recognize that in using study duration as the primary dependent variable for analysis, we are subjecting our results to the inherent variability of prospectively estimated study durations, which may differ greatly from actual study durations reported post trial. To address this limitation in the reliability of our data, we will monitor the status of all reviewed trials as they move toward completion and update our results to reflect any significant divergences between estimated and actual study duration.

Due to time and resource constraints, we did not perform an exhaustive search of all mHealth trials that had published either manuscripts or protocols in the literature during our 1-year search period. Our decision to have a sampling method solely focused on a single trials registry may have resulted in a biased identification of trials with more traditional positivist methods—this is also suggested by how the trials we reviewed were largely academically sponsored. We acknowledge that the trials registered on ClinicalTrials.gov do not make up the sum total of mHealth research. There is a large body of mHealth evaluative work that is not registered on ClinicalTrials.gov, notably apps that have engaged in usability testing and feasibility pilot studies but have not undergone formalized clinical research [22,40-44], as well as direct-to-consumer apps that publish evaluative reports of their in-house testing online but do not submit their work for review through formal research channels [45-47]. As such, our findings on the homogeneity of mHealth clinical trial methods are limited to trials registered on ClinicalTrials.gov. We aim to conduct a more systematic search of the mHealth literature and also search additional mobile app store catalogues (ie, Windows, Samsung, Blackberry) for publicly available trial apps in a future review to improve the representativeness of our findings.

### Conclusion

It is clear that mHealth evaluation methodology has not deviated from common methods, despite the issues raised. There is a need for clinical evaluation to keep pace with the rate and scope of change of mHealth interventions if it is to have relevant and timely impact in informing payers, providers, policy makers, and patients. To fully answer the question of an app’s clinical impact, mHealth researchers should maintain a reflexive position [35] and establish feasible criteria for rigor that may not ultimately result in a positivist truth but will drive an interpretive understanding of contextualized truth. As the mHealth field matures, it presents the challenge of establishing robust and practical evaluation methodologies that further foundational theory and contribute to meaningful implementation and actionable knowledge translation—all for optimized patient health and well-being.

## Abbreviations

ANOVA: analysis of variance
CEEBIT: Continuous Evaluation of Evolving Behavioral Intervention Technologies
GPS: global positioning system
MOST: Multiphase Optimization Strategy
RCT: randomized controlled trial
SMART: Sequential Multiple Assignment Randomized Trial
SMS: short message service
